# Natural Products for the Prevention and Treatment of Hangover and Alcohol Use Disorder

**DOI:** 10.3390/molecules21010064

**Published:** 2016-01-07

**Authors:** Fang Wang, Ya Li, Yu-Jie Zhang, Yue Zhou, Sha Li, Hua-Bin Li

**Affiliations:** 1 Guangdong Provincial Key Laboratory of Food, Nutrition and Health, School of Public Health, Sun Yat-Sen University, Guangzhou 510080, China; missingfeng@yeah.net (F.W.); saferide@126.com (Y.L.); zhyujie3@mail2.sysu.edu.cn (Y.-J.Z.); zhouyue3@mail2.sysu.edu.cn (Y.Z.); 2 School of Chinese Medicine, The University of Hong Kong, Hong Kong, China; lishasl0308@163.com

**Keywords:** natural product, hangover, alcohol use disorder, hepatoprotection

## Abstract

Alcoholic beverages such as beer, wine and spirits are widely consumed around the world. However, alcohol and its metabolite acetaldehyde are toxic and harmful to human beings. Chronic alcohol use disorder or occasional binge drinking can cause a wide range of health problems, such as hangover, liver damage and cancer. Some natural products such as traditional herbs, fruits, and vegetables might be potential dietary supplements or medicinal products for the prevention and treatment of the problems caused by excessive alcohol consumption. The aim of this review is to provide an overview of effective natural products for the prevention and treatment of hangover and alcohol use disorder, and special emphasis is paid to the possible functional component(s) and related mechanism(s) of action.

## 1. Introduction

Alcoholic beverages are widely consumed around the world. Alcohol consumption has both adverse and beneficial effects. The health effects of drinking depend on the quantity and pattern of alcohol consumption. Although several studies have showed that light to modest alcohol consumption (especially wine or beer) is linked to a decrease in cardiovascular events and total mortality [1,2], some studies indicated that the relationship between alcohol consumption and several cardiovascular diseases is uncertain or negative even at moderate intakes [3,4,5]. Furthermore, excessive alcohol consumption adversely affects human health.

Acute binge alcohol ingestion has been associated with hangover symptoms and even organ damage. In general, hangover is characterized by unpleasant physical and mental symptoms after alcohol consumption, such as dizziness, headache, fatigue and muscle pain [6,7]. In addition, hangover has adverse social and economical influence, such as a high incidence rate of traffic and violence accidents as well as decreased occupational skill and performance [8]. Symptoms of hangover seem to be the combined result of dehydration, hormonal alterations, dysregulated cytokine pathways, and the toxic effects of alcohol and acetaldehyde [9]. Excessive ingestion of alcohol, whether acute or chronic, is responsible for a tremendous disease and disorder, not only alcoholic hepatitis, cirrhosis and hepatocarcinoma, but also a series of other dysfunctions including pancreatitis, cardiomyopathy, hypertension, stroke, and fetal alcohol syndrome [10,11,12,13]. Excessive consumption of alcohol also results in damage to the central nervous system, such as polyneuritis, cerebellar degeneration, alcoholic dementia, pellagra encephalopathy, Marchiafava-Bignami and Wernicke-Korsakoff syndromes [14,15,16]. Moreover, epidemiological studies have identified chronic alcohol consumption as a significant risk factor for cancers of the upper aerodigestive tract (such as oral cavity, pharynx, larynx and esophagus) and liver [17]. Daily alcohol ingestion of more than 20.44 g was related with an increasing risk of both liver cancer incidence (hazard ratio (HR) 1.52, 95% CI 1.06–2.18) and liver disease mortality (HR 6.68, 95% CI 4.16–10.71) [12]. In addition, with long-term overconsumption of alcohol plus environmental stimuli, alcohol drinking may become habitual, which might be a risk factor for alcohol use disorder [18]. Alcohol use disorder is a devastating illness that affects a large population. It has been demonstrated that alcohol use disorder is the World’s third largest risk factor for disease and disability. It is estimated that overconsumption of alcohol causes 3.8% of all global deaths and 4.6% of global disability-adjusted life-years [19].

Alcohol metabolism proceeds via oxidative and non-oxidative pathways. The main processes of the oxidative pathway are mediated by alcohol dehydrogenase (ADH) and acetaldehyde dehydrogenase (ALDH), which transform alcohol into acetaldehyde and then to acetate, respectively [20]. Long-term chronic alcohol consumption reduced hepatic ADH and exacerbated the adverse reactions. Acetaldehyde, which is the first metabolite of alcohol oxidation, could lead to a series of unpleasant feelings such as nausea, vomiting, headache and fatigue [21]. Acetaldehyde is categorized as a group 2B carcinogenic substance by the World Health Organization International Agency for Research on Cancer, meaning it is possibly carcinogenic to humans [22].

Studies have showed that oxidative stress, much of it produced by activating NADPH oxidase, is a dominating mediator of a number of the pathogenic effects of excessive chronic alcohol consumption [23]. Cytochrome P450s, especially cytochrome P450 2E1 (CYP2E1), is also involved in the oxidation of alcohol. Reactive oxygen species (ROS), such as hydrogen peroxide and superoxide ions, generated by CYP2E1 are contributors to the pro-inflammatory profile of alcohol-related liver damage [24,25]. Alcohol consumption disturbs the balance between the pro- and anti-oxidant systems of the organism, so as to cause oxidative stress [26]. Free radicals or reactive oxygen species attack fats and proteins and rapidly enter cell membranes causing damage to the membrane, which leads to alcohol-induced oxidative tissue injuries. Therefore, effective antioxidant and anti-inflammatory drugs or foods might be useful for alleviating the harmful health consequences of excessive alcohol consumption [27,28,29,30].

Both behavioral approaches and pharmacological agents are current treatments for alcohol use disorder. The pharmacological treatment of patients with alcohol use disorder is very necessary in achieving the goal of alleviating the physical as well as the motivational aspects of the withdrawal syndrome, attenuating ongoing alcohol use disorders, reducing tolerance and preventing relapse. In brief, the pharmacological management of alcohol use disorders may be considered as two phases. The first phase is centered on detoxification and treatment of the acute abstinence syndrome while the second phase of treatment aims at preventing relapse. Three drugs approved by United State Food and Drug Administration are available for the treatment of alcohol use disorder, that is, disulfiram, naltrexone and acamprosate. However, most treatments have several shortcomings, such as neuritis, gastrointestinal (nausea) and central nervous system-related symptoms [31]. Thus, novel treatments are being developed and researched with the intention of improving effectiveness. Recent experimental evidences suggested that novel pharmacological approaches for treatment of hangover and alcohol use disorders may derive from natural products [32,33]. Several plant-derived compounds have been shown to significantly reduce alcohol intake, alcohol craving and withdrawal syndrome. The development of efficient medicines from natural products also exhibits expansive market prospects [34]. This paper gives an overview of natural products for prevention and treatment of hangover and alcohol use disorder to alleviate health burden of alcohol-induced disease and injury, with a special emphasis on their possible functional component(s) and related mechanism(s) of action.

## 2. Natural Products with Anti-Hangover Properties

Herbal therapies for hangover have been used for several centuries. Medicinal plants, fruits and vegetables are rich in antioxidants such as polyphenolic components, isoflavonids and vitamins, which could scavenge free radicals [35,36,37,38]. Previous rodent studies implicated oxidative stress as a key mediator of hangover syndrome, and demonstrated that various antioxidants could suppress the adverse events caused by alcohol exposure [39]. Several natural plants and products showed positive effects on alcohol metabolism in animal and human studies. They could upgrade the levels of ADH and ALDH in liver and decrease the concentration of alcohol in blood.

### 2.1. Pueraria Lobata

Kudzu (*Pueraria lobata*) is an important herb used for various diseases. Kudzu possesses the ability of ameliorating hangover symptoms and has been used for the treatment of chronic alcoholic liver injury in traditional Chinese medicine for a long time. In addition, it has been used to treat alcohol use disorder.

Two parts (roots and flowers) of *Pueraria lobata* are usually used in traditional medicine. The flowers have been used to treat the problems caused by alcohol drinking due to their ability to enhance acetaldehyde removal [40]. A clinical study suggested that *Puerana thomsonii* (one kind of the kudzu) had a certain stimulatory effect on the clearance of blood acetaldehyde in humans, which might reduce acetaldehyde toxicity and hangover symptoms such as flushing, palpitations, and headache [41]. Tectoridin, an isoflavone glycoside isolated from the flowers of *Pueraria lobata*, had hepatoprotective effects against alcohol-induced liver steatosis by significantly decreasing the levels of alanine aminotransferase (ALT), aspartate aminotransferase (AST) and triglyceride (TG) in serum, modulating the disturbance of peroxisome proliferators-activated receptor α pathway as well as ameliorating the hepatic mitochondria dysfunction in mice [42]. In addition, the flowers of kudzu exerted protective effects against alcohol-induced apoptosis in human neuroblastoma cells [43].

Meanwhile, the roots of *Pueraria lobata* showed inhibitory activity against mitochondrial ALDH2, and could increase the concentration of acetaldehyde in blood. Therefore, it could be used as an aversion therapy for alcohol use disorder [40]. The extract of Kudzu is a safe and effective product for alcohol use disorder. It is the only natural medication regarded by the National Institute on Alcohol Abuse and Alcoholism to treat alcohol use disorder [44]. In a clinical population study, kudzu treatment resulted in significant reduction in alcohol intake in a naturalistic setting. The number of beers consumed and the volume of each sip was decreased while the number of sips and the time to consume each beer was increased. There were no reported side effects of kudzu treatment [45]. In another study, 20 men participated in a placebo-controlled, double-blind design experiment, where kudzu extract (2 g) with an active isoflavone content of 520 mg, quickly reduced alcohol intake in a binge drinking paradigm [46].

**Figure 1:**
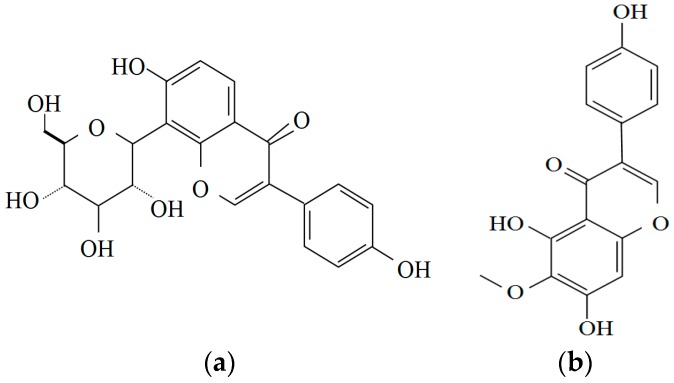
Structures of two bioactive components in Kudzu: (**a**) Puerarin; and (**b**) Tectoridin.

Puerarin and daidzein, two isoflavonoids isolated from the dried roots of *Pueraria lobata*, have been reported to be efficient in the treatment of various diseases [41]. Especially, puerarin has the potential of treating the alcohol use disorder through reducing the anxiogenic effects of alcohol withdrawal in rat. The social interaction and locomotor activity were increased after withdrawal from 17 days of alcohol (7%) diet [47]. In addition, puerarin reduced hepatotoxicity in CCl_4_-induced hepatic fibrosis and chronic alcoholic liver injury in rats via the following underlying mechanisms: (a) regulated enzymes (ALT, AST), albumin, and total protein in blood; (b) inhibited Kupffer cells activation and attenuated TNF-α/NF-κB pathway for anti-inflammation response; and (c) improved metabolic function in liver tissue [48]. Additionally, owing to the antioxidant ability of *Pueraria lobata*, the activity of superoxide dismutase (SOD) was increased and the level of malondialdehyde (MDA) was decreased in liver [48,49]. Therefore, the flowers of kudzu might have the ability to alleviate hangover and provide hepatoprotection while the root of kudzu was effective in reducing alcohol intake in several clinical population studies. The structures of puerarin and tectoridin are shown in Figure 1.

### 2.2. Fructus Evodiae

*Fructus evodiae* is a widely used herbal medicine in China with anti-inflammatory and analgetic activities. Dehydroevodiamine, evodiamine and rutaecarpine are the dominant bioactive constituents in *Fructus evodiae* [50]. The extract of *Fructus evodiae* could be used as a potential remedy for hangover symptoms induced by alcohol on mice by stimulating the expression of hepatic alcohol metabolizing and antioxidant enzymes [51]. The results showed that among all groups the plasma alcohol concentrations were the lowest in *Fructus evodiae* treated groups. Moreover, the expressions of liver alcohol metabolizing and antioxidant enzymes were also enhanced. The relative expression of ADH and Zn-Cu SOD increased more in treatment groups than that in positive controls. In another study, a water extract of *Fructus evodiae* possessed the ability to alleviate alcohol-induced gastric lesions in rats by strengthening the mucosal barrier integrity and increasing gastric mucosal nitric oxide synthesis [52]. Therefore, *Fructus evodiae* could be a candidate for the prevention and treatment of hangover and organ damage induced by alcohol through modulating alcohol metabolism and antioxidant enzymes in the liver.

### 2.3. Trigonela Foenum-Graecum

Seeds of fenugreek (*Trigonela foenum-graecum*) are reported to possess hepatoprotective activity. The aqueous extract of fenugreek seeds offers a striking protection against alcohol toxicity. Fenugreek seed polyphenolic extract (FPEt) acted as a protective agent against alcohol-induced hepatocyte abnormalities. The study showed that FPEt ameliorated the pathological liver changes and changed protein expression in Chang liver cells as well as improved the levels of antioxidant enzymes. The effects of FPEt were identical to those of the known hepatoprotective agent, silymarin. FPEt might exert cytoprotective effects by enhancing cellular redox status [53]. Treatment with FPEt restored the levels of markers of liver injury (AST, ALT, ALP, lactate dehydrogenase (LDH), bilirubin and GGT) and enhanced alcohol metabolizing and detoxification enzymes, as well as the electron transport component cytochrome-c reductase. After the intervention of FPEt in cells, the expression of ADH, ALDH, and CYP2E1 were upregulated, whereas the expression of cytochrome-c was downregulated in the alcohol-treated cells. Increased hepatocyte viability and reduced apoptotic nuclei were observed in FPEt-treated rats [54]. In addition, the expression of cellular heat shock proteins-HSP70, HSC70, HSC92, and mitochondrial protein mtHSP70 were produced in alcohol-treated Chang liver cells, which suggested a protective effect of FPEt [55]. Moreover, FPEt administration had a positive influence on both lipid profile and collagen properties in alcoholic liver disease. Treatment of alcohol-fed rats (200 mg/kg/day) with FPEt significantly reduced the levels of lipid peroxidation products and protein carbonyl content, as well as prevented the leakage of enzymatic and lipid peroxidation rise [56]. In a word, the FPEt increased the activities of antioxidant enzymes and enhanced the antioxidant properties, which could be the potential mechanisms of action in chronic alcohol-fed mice. The protective effect was possibly due to the bioactive antioxidants in fenugreek seeds such as polyphenols [57,58,59]. As a result, *Trigonela foenum-graecum* might have a positive influence on suppressing the abnormalities induced by alcohol in chronic alcohol liver diseases through its antioxidant properties.

### 2.4. Hovenia Dulcis

*Hovenia dulcis* are distributed throughout East Asia. The peduncles of *Hovenia dulcis*, which have been used as a traditional herbal medicine in China for a long time, contain abundant nutrients [60]. It possesses free radical scavenging ability and could enhance physical activity [61,62]. Owing to its hepatoprotective ability, it has been used for the treatment of liver diseases and alcohol toxicity. The effective constituents might be heteropolysaccharides, which mainly consist of rhamnose, arabinose, galactose and galacturonic acid [63]. Treatment with peduncles of *Hovenia dulcis* decreased the serum levels of ALT and AST, decreased the liver malondialdehyde (MDA) level and restored liver antioxidant enzymes such as SOD, glutathione S-transferase (GST) and glutathione peroxidase (GSH) in alcohol-induced liver injury mice [63]. In addition, administration of *Hovenia dulcis* extract increased ADH activity in alcohol-ingesting mice and stimulated alcohol metabolism [64]. Dihydromyricetin (DHM), a flavonoid separated from *Hovenia dulcis*, was identified to interact with γ-aminobutyric acid receptors and block alcohol intoxication and withdrawal signs in rats such as tolerance, increased anxiety, and seizure susceptibility. DHM could remarkably reduce alcohol digestion in a voluntary alcohol intake paradigm in rats. At the cellular level, DHM treatment antagonized potentiation of GABA_A_ receptors and plasticity. Therefore, DHM could be used as a therapeutic candidate for alcohol use disorders [44,65]. In conclusion, *Hovenia dulcis* could be a therapeutic candidate for alcohol-induced liver injury and alcohol use disorders.

### 2.5. Pyrus Pyrifolia

*Pyrus pyrifolia* (Korean pear) has been used as a prophylactic agent for alleviating alcohol hangover. Polyphenols are the major bioactive components of *Pyrus pyrifolia* [66]. Lee *et al.* [67] performed a randomized single blind crossover trial with 14 healthy young men to test the effects of Korean pear juice on hangover. The total and average of hangover severity were decreased to 16% and 21% by *Pyrus pyrifolia* juice after the alcohol consumption, respectively (*p* < 0.05). Impaired memory and sensitivity to light and sound were significantly improved among the subjects. In addition, the pear juice treatment decreased the levels of blood alcohol (*p* < 0.01). The results have showed that Korean pear stimulated the activities of both ADH and ALDH and decreased the blood alcohol level in ALDH2 genotype. However, the pear could increase the concentration of acetaldehyde in blood in ALDH2 deficient mice, without affecting the concentration of acetaldehyde in ALDH2 normal mice. These enzyme stimulations might be the main mechanism of the alcohol detoxification effects Korean pear for [68]. Therefore, Korean pear juice could alleviate hangover, and its detoxification of alcohol seemed to be related to the genetic variation of ALDH2. The results suggested that human ALDH2 polymorphisms could lead to individual variations on alcohol detoxification. Hence, *Pyrus pyrifolia* might be a useful and effective food supplement in alleviation of hangover and detoxification of alcohol through stimulating the activities of both ADH and ALDH.

### 2.6. Mangifera Indica L.

Mango (*Mangifera indica* L.) is a widely consumed tropical fruit. It is rich in polyphenolic compounds which could protect from several diseases. Mango fruit intake provides antioxidants that may act in a synergistic way with other foods to offer protection [69]. Kim *et al.* [70] confirmed that mango flesh and peel had ameliorating effects on plasma alcohol levels and increased the activities of ADH and ALDH in mice. A loading plot indicated that several compounds in mango fruit, such as fructose and aspartate, might enhance alcohol metabolism. As a result, mango flesh and peel could be the source of functional foods with the intention of decreasing plasma alcohol level after excessive alcohol intake.

### 2.7. Diospyros Kaki Thunb.

Persimmon (*Diospyros kaki* Thunb.) is a fruit containing high levels of phenolics that could be used for making vinegar. Administration of persimmon-vinegar provided a protection to metabolic disorders induced by chronic alcohol ingestion in rats. It obviously decreased serum triglyceride, total cholesterol and liver total cholesterol levels. The liver non-esterified carnitine level was increased in the persimmon-vinegar-administered groups, which means a protection of lipid oxidation. In addition, the blood alcohol concentration was the lowest in high-dose persimmon-vinegar-administered group [71]. In addition, the administration of the extract from leaf and fruit of persimmon suppressed acute alcohol-induced hepatotoxicity in mice. The alcohol metabolism was accelerated by increasing alcohol-metabolizing enzyme activities and activating the antioxidative enzyme system against oxidative stress as well as decreasing fat accumulation [72]. Therefore, the extract from fruit and leaf of persimmon might have the ability to improve alcohol metabolism and liver lipid profile due to its antioxidant components such as flavones and phenolics.

### 2.8. Thymus Vulgaris

The extracts of thyme (*Thymus vulgaris*) have detoxifying and antioxidant effects. The leafy parts of thyme and its essential oil have been widely used in food for flavor, aroma and preservation and also in traditional medicines [73]. The essential oil of thyme has showed free radical scavenging and antibacterial activity [74], and it could detoxify alcohol toxicity. Thymol was the major component (44.4%–58.1%), followed by *p*-cymene (9.1%–18.5%), γ-terpinene (6.9%–18.9%), and carvacrol (2.4%–4.2%) in the tested oil samples [75]. The water extract of thyme possessed the ability of detoxifying the injuries of alcohol on liver and brain in mice. It could decrease nitric oxide and MDA level in liver and brain, and increase the total antioxidant capacity and GPx activity [76]. Therefore, *Thymus vulgaris* was recommended to treat alcohol toxicity through its potent antioxidant properties.

### 2.9. Zingiber Officinale

Ginger (*Zingiber officinale*) has been used as an important ingredient in cooking and traditional herbal medicine for a long time. It exhibits antioxidant potential and hepatoprotective activity. 6-Gingerol as the major bioactive constituent of ginger could efficiently scavenge various free radicals [77]. The antioxidant compounds of ginger may modulate the oxidative stress induced by alcohol. SOD, ascorbic acid, and GSH levels were decreased, and GST activity was increased in alcohol treated rats. However, after treatment with the extract of ginger, these parameters came to normal [78].

Owing to the antioxidant effect of ginger, *Zingiber officinale* is recommended to be used as natural product to treat alcohol toxicity. The water extract of ginger could decrease the levels of both l-γ-glutamyl transpeptidase and butyryl cholinesterase [76]. A formula (KSS formula) consisting of pith of citrus tangerine, the rhizome of *Zingiber officinale*, and brown sugar has been traditionally used in China for the treatment of discomfort after excessive alcohol ingestion. In a clinical effectiveness evaluation study, the hangover symptoms such as nausea, vomiting and diarrhea were alleviated after administration of formula in scheduled prophylactic doses [79].

Excessive alcohol consumption caused alcoholic fatty liver disease (AFLD). The ginger essential oil and citral exhibited hepatoprotective activity against AFLD in mice. The amounts of metabolites in serum such as d-glucurono-6,3-lactone, glycerol-3-phosphate, pyruvic acid, lithocholic acid, 2-pyrocatechuic acid, and prostaglandin E_l_ increased after alcohol administration, but the levels were recovered in treatment groups [80]. Therefore, ginger could be used as a candidate to the prevention and treatment of hangover and organ damages induced by overconsumption of alcohol through its antioxidant action.

### 2.10. Asparagus Officinalis

*Asparagus officinalis*, a popular vegetable, is consumed widely and has long been used as an herbal medicine to several diseases. *Asparagus officinalis* is applied for alleviating hangover and protecting liver cells from alcohol toxic. The dietary fiber and flavonoids of *Asparagus officinalis* improved the plasma lipid profile and reduced liver oxidative damage in hypercholesterolemia mice model [81]. Kim *et al.* [82] analyzed the constituents of the young shoots and the leaves of asparagus, the result showed that the amino acid and inorganic mineral contents were higher in leave than in shoots. They also demonstrated that cellular toxicity induced by alcohol was relieved after treatment with the extracts of *Asparagus officinalis* leave and shoots. Additionally, the activities of two key enzymes that metabolize alcohol, ADH and ALDH, increased after treatment of leaf and shoot extracts [82]. As a result, *Asparagus officinalis* might be used as a natural product to prevent and treat hangover through increasing alcohol metabolism by upregulating the activities of ADH and ALDH.

### 2.11. Oenanthe Javanica

Water dropwort has long been used for the treatment of inflammatory diseases, including hepatitis [83]. The extract of water dropwort (*Oenanthe javanica*) is effective in alleviating alcohol intoxication by accelerating alcohol metabolism. Using New Zealand white rabbit and ICR mice as animal models, Kim *et al.* [84] discovered that after the treatment with hot-water extract of water dropwort in rabbit, the plasma alcohol levels were rapidly decreased, identical to treatment of standard drug, metadoxine. Specifically, the *n*-butanol fraction of hot-water extract was the strongest in eliminating plasma alcohol in ICR mice. Hot-water extract cleared 44% of the plasma alcohol while the *n*-butanol fraction eliminated around 70% in mice. Alcohol removal behaved in a dose-dependent manner in the range of 50–200 mg/kg in the *n*-butanol fraction. Caffeic acid in water dropwort might be a contributor to the protective action from oxidative stress-induced liver damage [85,86]. Therefore, water dropwort might be another potential candidate to treat hangover through accelerating alcohol metabolism.

### 2.12. Opuntia Ficus-Indica

*Opuntia ficus-indica* (pear cactus) is a xerophyte plant that belongs to the Cactaceae family. The cactus pears have strong antioxidant properties due to its high contents of polyphenolics, flavonoids, betacyanin, betaxanthin, taurine and ascorbic acid [87]. The extract of the *Opuntia ficus-indica* (OFI) had a modest effect on alleviating hangover symptoms by inhibiting the production of inflammatory mediators, because the symptoms of hangover are partly due to the activation of inflammation. In a double-blind, placebo-controlled, crossover trial, 64 healthy, young adult volunteers were randomly assigned to receive OFI (1600 IU) and identical placebo 5 h before alcohol consumption. The results showed that three of the nine symptoms—nausea, dry mouth, and anorexia—were significantly reduced by OFI. The risk of a severe hangover (greater than or equal to 18 points) was decreased markedly (odds ratio, 0.38; 95% confidence interval, 0.16–0.88; *p* = 0.02) [88,89].

The prickly pear juice possessed the ability against alcohol-induced liver injury in rats due to their capacity to scavenge free radicals or to enhance the endogenous antioxidants activities. Chronic alcohol administration (3 g/kg) to Wistar rats for 90 days, significantly increased the liver lipid and protein oxidation (*p* < 0.01), reduced the GSH content and the activities of liver antioxidant enzymes such as SOD, catalase, GSH and conversely elevated the liver injury biochemical markers such as AST, ALT, ALP, γ-GST, LDH, cholesterol, triglycerides and caused severe histopathologic injuries. Conversely pre-treatment with prickly pear juice (20 and 40 mL/kg, orally) in alcohol-fed rats, decreased liver lipid and protein oxidation and changed histopathologic injury, as well as inhibited the alterations of antioxidant enzymes and the release of biochemical markers [90]. Therefore, *Opuntia ficus-indica* might be effective in treating hangover and protecting liver from alcohol toxicity due to its anti-inflammatory and strong antioxidant properties.

### 2.13. Panax Ginseng

Asian ginseng (*Panax ginseng*) has therapeutic potential for the treatment of alcohol toxicity and as an anti-hangover agent. Ginseng shows positive effects on alcohol metabolism and relieved hangover symptoms. In addition, it also has protective effects to alcohol-induced toxicity in major organs in animals such as reproduction and gastric.

Red ginseng showed positive effects on hangover symptoms. Lee *et al.* [91] investigated the effects of red ginseng on relieving alcohol and hangover symptoms in 25 healthy men in a randomized crossover study. After ginseng intervention, the blood alcohol levels and expiratory air-alcohol levels decreased and acetaldehyde levels slightly increased compared with the control. The anthropometric parameters and hangover symptoms were also decreased. In another study, ginsenoside-free fraction from steam-dried ginseng berries have the abilities of promoting alcohol metabolism and scavenging free radicals *in vitro* and *in vivo* by stimulating primary enzymes (ADH, ALDH CYP2E1, and catalase). It was assumed that linoleic acid might be the most active ingredient [92].

Red ginseng extract also has protective effects from alcohol-induced male reproductive toxicity. There was a significant reduce in sperm motility and progressiveness in mice treated with alcohol for 5 weeks, while administration of red ginseng extract appeared to minimize the harmful effects of alcohol-induced toxicity on male fertility [93]. Besides, Haron *et al.* [94] using Japanese ricefish (*Oryzias latipes*) as an animal model of fetal alcohol spectrum disorder, identified that *Panax ginseng* could attenuate alcohol toxicity in embryogenesis. *Panax ginseng* may provide a protection to alcohol-induced trabecular cartilage deformities in the neurocranium in 1–3 day post fertilization group embryos. Meanwhile, black ginseng has a protective effect on alcohol-induced teratogenesis through the augmentation of antioxidative capacity in mouse embryos. The morphological scores were significantly increased compared with the control. The mRNA levels of GPx, phospholipid hydroperoxide, and selenoprotein were significantly improved compared with the alcohol-treated embryos [95]. Moreover, ginseng had protective effects from alcohol-induced gastric damages in rats. Significant induction of cytoprotective heat-shock proteins HSP27 and HSP70 was found in the ginseng-administrated rats, which suggested that the restoration of these proteins might contribute to preventing alcohol-induced gastric injuries [96]. Therefore, red ginseng could be used as potential treatment of hangover and alcohol-induced reproductive and gastric toxicity due to its antioxidant activity.

## 3. Natural Plants for Alcohol Use Disorder

Alcohol use disorder involves repeated alcohol use which leads to tolerance, alcohol withdrawal syndrome, physical and psychological dependence as well as compulsive and uncontrolled consumption of alcoholic beverages. The most important purpose of treating alcohol use disorder is centered on reducing alcohol withdrawal syndrome and improving alcohol drinking behaviors. Currently there is no effective therapeutic agent without side effects for alcohol use disorder. Several drugs are available to treating the alcohol use disorder, such as disulfiram (aldehyde dehydrogenase inhibitor), naltrexone (opioid antagonist), topiramate (GABAergic anticonvulsant) and acamprosate (NMDA/glutamate receptor modulator) [97,98,99]. They can reduce voluntary alcohol intake and alcohol cravings. However, compared with natural products, they have some serious side effects (such as ataxia, impaired attention and bad consciousness). Herbal remedies for alcohol use disorder have been in use in China for several centuries. Kudzu (*Pueraria lobata*), mentioned above, could be used for the treatment of alcohol use disorders, and puerarin isolated from kudzu could reduce the anxiogenic effects of alcohol withdrawal [47]. Dihydromyricetin, a flavonoid purified from *Hovenia dulcis* could be another therapeutic candidate for alcohol use disorder [65]. *Hypericum perforatum* and *Salvia miltiorrhiza* could be potential natural products to treat alcohol use disorder and will be discussed below, while *Scutellaria baicalensis* is important in the treatment of liver disease.

### 3.1. Hypericum Perforatum

**Figure 2:**
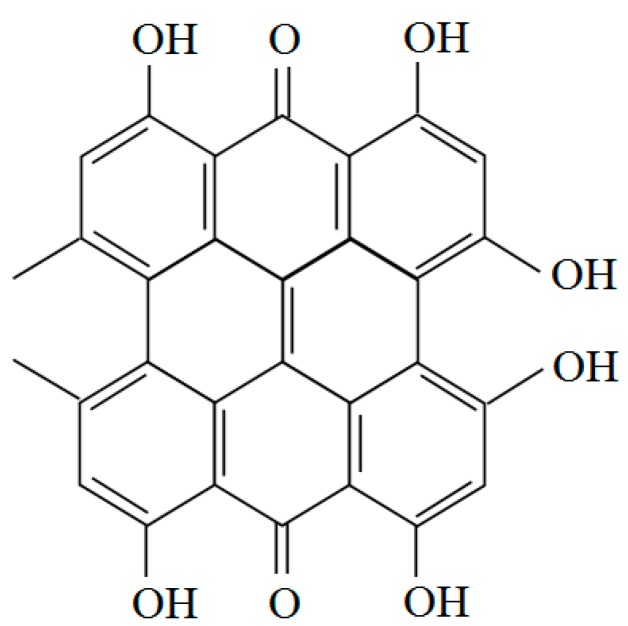
Structure of hypericin.

*Hypericum perforatum* is usually called St. John’s Wort. The extract of *Hypericum perforatum* (HPE) is widely used for the treatment of affective disorders [100]. It could reduce voluntary alcohol intake in Marchigian Sardinian alcohol-preferring (msP) rats and act synergistically with opioid receptor antagonists to further reduce alcohol consumption. The effect on alcohol intake of the combined treatment remained stable during the 12 days of chronic treatment. The food intake was slightly reduced, while no change on body weight, was observed compared with the control. The whole treatment was without development of tolerance [101]. HPE (50 and 100 mg/kg) produced positive inhibitory effects on tremor and audiogenic seizures during the withdrawal period in alcohol-preferring rats. These results suggest that HPE could have some beneficial effects on alcohol withdrawal syndrome in people [102]. *Hypericum perforatum* exhibits remarkable antioxidant potential due to high content of phenolic compounds, especially flavonoids, hyperforin and hypericin [103]. Hyperforin has NMDA-receptor-antagonistic and potential neuroprotective effects *in vitro* which might provide therapeutic effectiveness in the relapse prevention of alcohol use disorders [103]. In addition, hyperforin could reduce alcohol intake more effectively than hypericin in alcohol-dependent mice [104]. The CO_2_ extract of hypericum has been also shown to mediate alcohol intake in msP rats. Animal tests verified that the CO_2_ extract with 24.33% hyperforin and a very low content of hypericin inhibited alcohol intake more potently than the alcoholic extract containing 0.3% hypericin and 3.8% hyperforin. Hyperforin might thus play an important role in reducing alcohol intake. Neurochemical mechanisms are responsible for the reduction of alcohol intake and the antidepressant-like effect of HPE [105]. *Hypericum perforatum* markedly reduced alcohol intake in msP rats, and its effect was behaviorally selective. In other studies, the GABA_A_ receptor antagonist bicuculline and the GABA_B_ receptor antagonists CGP-36742 did not modify the effect of CO_2_ extract of hypericum. These results indicated that the inhibitory effects of HPE on alcohol intake are not mediated by GABA agonist actions [106,107]. In conclusion, *Hypericum perforatum* might be effective to improving alcohol drinking behaviors, although more efforts are needed to clarify the specific mechanisms. The molecular structure of hypericin is shown in Figure 2.

### 3.2. Salvia Miltiorrhiza

Danshen, the dried roots of *Salvia miltiorrhiza*, is a classical herb with over 1000 years of clinical application [108]. It has many biological and pharmaceutical activities such as antioxidant, anti-inflammatory and anti-apoptotic properties. The extracts of *Salvia miltiorrhiza* could reduce voluntary alcohol intake and maintenance of alcohol drinking behavior in Sardinian alcohol-preferring (sP) rats. Two types of major bioactive components in *Salvia miltiorrhiza* are associated with its activities, including water soluble phenolic acids and lipophilic diterpenoid quinines [109].

Extracts from the roots of *Salvia miltiorrhiza* could reduce alcohol intake in sP rats. Intragastric administration of 200 mg/kg extract reduced alcohol intake by 40% and preference throughout a 4 day treatment of a 2-bottle free-choice regimen. Intragastric administration reduced blood alcohol levels by 60%. A possible mechanism was that *Salvia miltiorrhiza* curbed alcohol absorption from the gastrointestinal tract [110]. Pure miltirone, one of the possible bioactive components of *Salvia miltiorrhiza*, reduced alcohol intake in alcohol-experienced rats and delayed intervals of acquisition of alcohol-drinking in alcohol-naive rats in 2-bottle “alcohol (10%, *v*/*v*) *versus* water” choice regimen. The alcohol levels in blood were markedly reduced while the severity of alcohol withdrawal syndrome in alcohol-dependent rats was not attenuated with the intervention of *Salvia miltiorrhiza* extracts [111]. IDN 5082, a standardized extract of *Salvia miltiorrhiza*, could delay the acquisition of alcohol drinking behavior in rats. The reduction in alcohol intake was compensated by an increase in water intake [112]. In addition, the IDN 5082 possessed the anti-relapse properties in alcohol preferring rats through complete suppressing of the extra amount of alcohol consumed during the first hour of re-access to alcohol after 7 days of deprivation [113]. Another study has demonstrated that proper vehicle such as polysorbate 80 to form micelles with the active ingredient(s) of the *Salvia miltiorrhiza* might contribute to reducing effect on alcohol intake in sP rats [114].

**Figure 3:**
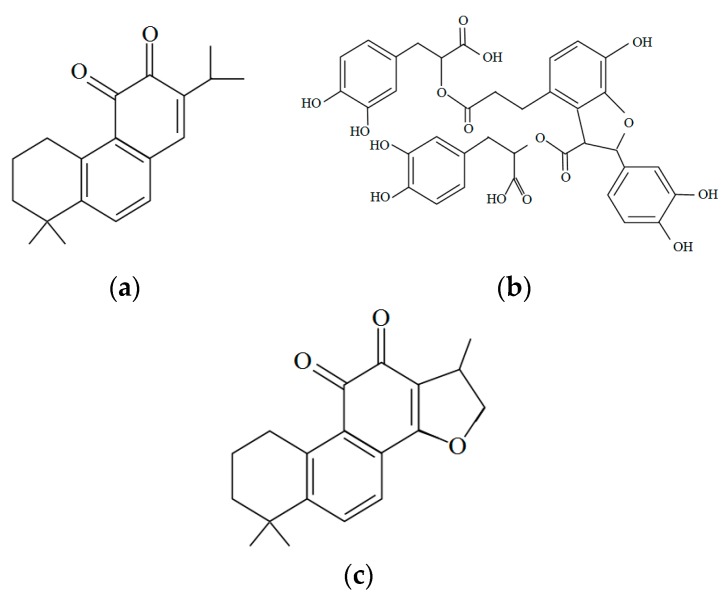
Structures of several bioactive components in *Salvia miltiorrhiza*: (**a**) Miltirone; (**b**) Salvianolic acid B; (**c**) Cryptotanshinone.

Salvianolic acid B (SalB) is an important bioactive component separated from the *Salvia miltiorrhiza*, which could attenuate acute alcohol-induced hepatocyte apoptosis in rats through SIRT1-mediated deacetylation of p53 pathway. Pretreatment with SalB significantly reduced alcohol-induced elevation in aminotransferase activities, decreased hepatotoxic cytokine levels such as interleukin-6 (IL-6), and increased the antioxidant enzyme activity. Moreover, SalB pretreatment inhibited the increase in NF-*κ*B, cleaved caspase-3 and decrease in B-cell lymphoma-extra large (Bc1-xL) caused by alcohol exposure, as well as notably increased the expression of SIRT1 and blocked Sa1B-induced acetylation of p53 down-regulation [115]. Several other compounds from *Salvia miltiorrhiza*, such as magnesium lithospermate B, cryptotanshinone, also could be potential effective for liver diseases caused by alcohol because they possessed anti-apoptosis and potential anti-inflammatory activities, and could be used for the treatment of NAFLD and liver cancers [116,117,118,119,120]. Therefore, *Salvia miltiorrhiza* might be a potential candidate for treating alcohol use disorder through reducing alcohol intake and preventing relapse, and protecting from alcohol induced liver damages through its anti-inflammatory activities. The structures of miltirone, salvianolic acid B and cryptotanshinone are shown in Figure 3.

### 3.3. Scutellaria Baicalensis

*Scutellariae Radix*, the root of *Scutellaria baicalensis*, is a Chinese herb widely used for the treatment of liver disease and cancer, as well as improving immune capacity [121]. Long-term liver damage and the wound-healing process resulted in liver fibrosis, in which the hepatic stellate cell (HSC) played a key role during fibrogenesis. The extract of *Scutellaria baicalensis* could prevent hepatic fibrosis by promoting ERK-p53 pathways, which may in turn cause G_2_/M cell cycle arrest and activate the Caspase system, finally resulting in apoptosis of HSC-T6 cells [122]. As a result, *Scutellaria baicalensis* might be beneficial for the amelioration of liver fibrosis.

**Figure 4:**
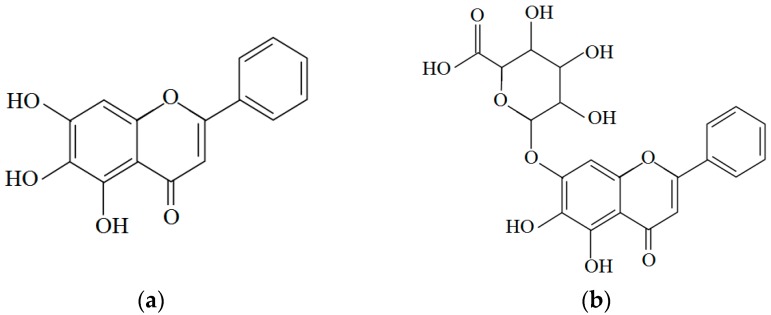
Structures of two bioactive components in *Scutellaria baicalensis*: (**a**) Baicalein; (**b**) Baicalin.

The main bioactive components of *Scutellaria baicalensis* are baicalein, baicalin and wogonin. Wogonin showed anti-inflammatory activity [123]. Baicalein possessed anti-hepatocellular carcinoma activity *in vitro* and *in vivo*. Baicalein at the concentrations of 40–120 μM exerted cytotoxicity to three hepatocellular carcinoma cell lines but with little cytotoxicity to a normal liver cell line *in vitro* [124]. In addition, baicalin has potential beneficial effects on ischemia/reperfusion (I/R) injury in alcoholic fatty liver. I/R-induced hepatocellular damage was attenuated by inhibiting TLR4-mediated inflammatory [125]. Moreover, baicalin had been found to prevent liver injury in several animal hepatitis models. Wan *et al.* [126] have proved that baicalin could effectively prevent LPS/D-GalN-induced liver injury in mice by suppressing NF-*κ*B activity, reducing TNF-α production. The underlying mechanism may be related to up-regulation of HO-1 protein activity. The structures of baicalein and baicalin are shown in Figure 4.

### 3.4. Rhizoma Coptidis

*Rhizoma coptidis* has a long history of clinic use in traditional Chinese medicine. It is effective in the treatment of gastrointestinal dysfunctions, including diarrhea, dysentery, and inflammation. Berberine is an isoquinoline alkaloid that is regarded as the major active constituent of *Rhizoma coptidis*. It has property of modulating several neurotransmitter systems, especially in alcohol use disorder. In an alcohol withdrawal-induced hyperexcitability paradigm of mice, acute and chronic administration of berberine (10 and 20 mg/kg) dose-dependently attenuated alcohol withdrawal-induced hyperexcitability signs, the effects were identical to diazepam (1.25 and 2.5 mg/kg). The major mechanism might be through its neuromodulatory action [127]. In addition, berberine attenuated alcohol-induced rewarding effects in mice through reducing locomotor stimulant effect and expression of sensitization to locomotor stimulant effect of acute alchol, reduced the induction and expression of alcohol-induced conditioned place preference as well as reducing preference to alcohol drinking over water [128]. Moreover, berberine had a positive effect on gastrointestinal injury induced by overconsumption of alcohol *in vivo* and *in vitro*. The results showed that berberine inhibited increases of alcohol-induced TNFα and IL-1β expression in gastrointestinal mucosa as well as upstream signals TLR2 and TLR4, and regulated cytokines [129]. Therefore, *Rhizoma coptidis* might reduce alcohol intake through its neuromodulatory action.

### 3.5. Other Natural Products for Alcohol Use Disorder

*Levo*-tetrahydropalmatine (L-THP), a derived compound from *Stephania ambigua* and *Corydalis teranda*, could regulate alcohol drinking in C57BL/6J mice using a 2-bottle drinking choice experiment. A single injection of L-THP increased active phosphorylated forms of PKA, AKT and ERK in the caudate-putamen. The reduction of alcohol drinking by L-THP treatment was possibly associated with dopamine D2 receptors-mediated PKA signaling in the caudate-putamen [130].

Brucine from the seeds of *Strychnos nux-vomica* L. is a glycine receptor antagonist. It selectively decreased alcohol consumption with minimal adverse effects in rats. The results showed that the treatment of brucine decreased the alcohol intake associated with a compensatory increase of water intake and unchanged daily total fluid intake and body weight in 2-bottle-choice drinking paradigm. Meanwhile, brucine suppressed the deprivation-induced elevation of alcohol consumption [131].

Ibogaine, a natural alkaloid, isolated from the root bark of *Tabernanthe iboga*, has been reported to markedly reduce voluntary alcohol intake in alcohol-preferring rats in 2-bottle choice and operant self-administration paradigms [132]. The possible mechanism was mediated by the glial cell line-derived neurotrophic factor (GDNF) in the ventral tegmental area [133]. Due to its side effects, however, ibogaine is not used clinically.

The leaves of *Jodina rhombifolia* are utilized to prevention of alcohol use disorder in Argentine folk medicine. Repeating administration of *Jodina rhombifolia* lyophilized extract markedly reduced alcohol voluntary intake in rats in self-administration model of 10 consecutive days, and the treatment was without apparent side-effects [134,135].

Finally, some natural products for the prevention and treatment of hangover and alcohol use disorder are summarized in Table 1.

**Table 1:** Natural products for the prevention and treatment of hangover and alcohol use disorder.

Natural Products	Bioactive Components	Part of Plant Used	Subjects	Biological Effects and Molecular Mechanism(s)	Reference
*Pueraria lobata*	Tectoridin	Dried flower	Humans	Reduced hangover symptoms by promoting the elimination of blood acetaldehyde	[41]
			Mice	Suppression of alcohol-induced liver steatosis by modulating the disturbance of the peroxisome proliferator-activated receptor α pathway and ameliorating mitochondrial function	[42]
			Cells	Suppression of alcohol-induced apoptosis in human neuroblastoma cells	[43]
	Puerarin and daidzein	Root	Humans	Reduced alcohol intake in a naturalistic setting	[45]
			Humans	Reduced alcohol consumption in a binge drinking paradigm	[46]
			Rats	Reduced anxiogenic effects of alcohol withdrawal via increased social interaction and locomotor activity	[47]
			Rats	Mitigation of liver damage (AST, ALT, GGT) and lipid deposition induced by chronic alcohol intake as well as TNF-α release, protein expression of endotoxin receptors	[48]
*Fructus evodiae*	Dehydroevodiamine, evodiamine and rutaecarpine	Dried and unripe fruit	Mice	Alleviation of hangover through stimulating the expression of hepatic alcohol metabolizing and antioxidant enzymes	[50]
			Rats	Prevention of alcohol-induced gastric mucosal lesions by strengthening the mucosal barrier integrity and increasing gastric mucosal nitric oxide (NO) synthesis	[51]
*Trigonela foenum-graecum*	Polyphenols	Seeds	Cells	Prevention of the toxic effects of alcohol through increased cell viability, reduced lactate dehydrogenase leakage and normalized GSH/GSSG ratios	[53,55]
			Rats	Suppressed alcohol-induced abnormalities in the liver through restoration of liver enzymes, ADH and ALDH activities	[54]
			Rats	Suppression of alcohol toxicity through prevention of enzymatic leakage, and improved lipid profiles	[56,57,58]
*Hovenia dulcis*	Heteropolysaccharides	Peduncle	Mice	Suppression of acute alcohol-induced liver injury through decreased serum levels of AST, ALT and liver MDA, and restored liver SOD, GST and GSH	[63,64]
	Dihydromyricetin		Rats	Reduced alcohol consumption in an intermittent voluntary alcohol intake paradigm	[65]
*Pyrus pyrifolia*	Polyphenols	Fruit	Humans	Alleviation of alcohol hangover through lowered blood alcohol levels and modifed genetic variation of ALDH2	[67]
			Mice	Alleviation of alcohol hangover through decreased blood alcohol levels	[68]
*Mangifera indica* L.	Polyphenols	Flesh and peel	Mice	Decreased plasma alcohol levels and increased activities of ADH and ALDH	[70]
*Diospyros kaki* Thunb.	Flavones and phenolics	Vinegar from fruit	Mice	Prevention of metabolic disorders induced by alcohol through blood alcohol clearance and decreased triglyceride and total cholesterol levels	[71]
		Leave and fruit	Mice	Prevention of hepatic injury by accelerating alcohol metabolism, activating the antioxidative enzyme system and decreasing fat accumulation	[72]
*Thymus vulgaris*	Essential oil	Leave	Mice	Amelioration of liver and brain alcohol injuries through decreased NO and MDA levels and increased the total antioxidant capacity and GPX activity	[76]
*Zingiber officinale*	6-gingerol	Rhizome	Mice	Amelioration of liver and brain alcohol injuries through decreasing L-γ-glutamyl transpeptidase and butyryl cholinesterase	[76]
			Humans	Decreased signs and symptoms of alcohol hangover	[79]
	Essential oil and citral		Mice	Hepatoprotective property against AFLD by decreasing levels of D-glucurono-6,3-lactone, glycerol-3-phosphate, and pyruvic acid in serum	[80]
*Asparagus officinalis*	Flavonoids	Shoots and the leaves	Cells	Alleviation of alcohol toxicity by upregulating the activities of ADH and ALDH	[82]
*Oenanthe javanica*	Caffeic acid	Leave and stem	Rats and mice	Alleviation of alcohol intoxication by accelerating alcohol metabolism	[84]
*Opuntia ficus-indica*	Flavones and phenolics	Cladode	Humans	Reduced hangover symptoms by inflammatory mediator production inhibition	[88,89]
			Rats	Suppression of liver damage induced by alcohol through ending free radical chain reactions or enhancing the endogenous antioxidant activities	[90]
*Panax ginseng*	Ginsenosides	Root	Humans	Relief from hangover symptoms through reduced expiratory and plasma alcohol levels and hangover severity	[91]
	Linoleic acid		Mice	Alleviation of hangover through reduced alcohol and acetaldehyde levels and enhanced ADH and ALDH activities	[92]
			Mice	Suppressed alcohol-induced toxicity on male fertility	[93]
			Ricefish	Suppressed alcohol-induced toxicity on embryogenesis	[94]
			Mouse embryos	Suppressed alcohol-induced toxicity in the neurocranium through its effects on antioxidant activity	[95]
			Rats	Suppressed alcohol-induced toxicity in the gastric system via the restoration of heat-shock proteins	[96]
*Hypericum perforatum*	Hypericin and hyperforin	Leave and flowering tops	Rats and mice	Reduced voluntary alcohol intake in acute and chronic alcohol treatment through neurochemical mechanisms	[101,104,105]
			Rats	Attenuated alcohol withdrawal syndrome by inhibition of the effects on tremors and audiogenic seizures	[102]
*Salvia miltiorrhiza*	Miltirone	Root	Rats	Reduced alcohol intake and blood alcohol levels through curbing alcohol absorption	[110]
			Rats	Reduced alcohol intake and delayed acquisition of alcohol-drinking behavior	[111]
	Idn 5082		Rats	Delayed acquisition of alcohol drinking behavior, and relapse prevention by suppressing the extra alcohol consumption after deprivation	[112,113]
	salvianolic acid B		Rats	Attenuation of acute alcohol-induced hepatocyte apoptosis through SIRT1-mediated deacetylation of the p53 pathway	[115]
*Scutellariae Radix*	Baicalein, baicalin and wogonin	Root	Rats	Attenuated liver fibrosis through liver sinusoidal endothelial cell activation and HSC migration	[121]
*Rhizoma coptidis*	Berberine	Rhizome	Rats	Reduced alcohol intake and withdrawal induced hyperexcitability through its neuromodulatory action	[127,128]
			Rats	Attenuated acute alcohol-induced gastrointestinal mucosa damage through regulation of cytokines	[129]
*Stephania ambigua* and *Corydalis teranda*	*Levo*-tetrahydro-palmatine		Rats	Reduced alcohol intake through dopamine D2 receptor-mediated PKA signaling in caudate-putamen	[130]
*Strychnos nux-vomica* L.	Brucine	Fruit	Rats	Decreased alcohol consumption through glycine receptor antagonist	[131]
*Tabernanthe iboga*	Ibogaine	Root	Rats	Reduced alcohol intake through mediation of glial cell line-derived neurotrophic factor	[132]
*Jodina rhombifolia*	Unclear	Leave	Rats	Reduced alcohol intake without tolerance and apparent side-effects	[134,135]

## 4. Conclusions

Overconsumption of alcoholic beverages is a well-recognized contributor to a variety of health problems, and may cause function disorders of major organs such as liver, brain, heart, lung and prostate. Several natural products have shown effective protection against alcohol-induced injuries and significant attenuation of hangover symptoms in several animal models and limited human tests The alcohol levels in blood were reduced, the hangover symptoms scores were lowered and the biochemical marks of liver injury were restored with natural plant treatments, and the mechanisms of action are mainly antioxidative and anti-inflammation. In addition, several natural products could be effective in reducing the voluntary alcohol intake, improving alcohol drinking behaviors and attenuating withdrawal syndromes of alcohol use disorder. Natural products have shown wide prospects for the prevention and treatment of hangover and alcohol use disorder. In the future, more bioactive compounds in plants (especially medicinal plants, fruits and vegetables) should be separated and identified, and the mechanisms of action should be studied further.
